# A CRISPR knockout negative screen reveals synergy between CDKs inhibitor and metformin in the treatment of human cancer in vitro and in vivo

**DOI:** 10.1038/s41392-020-0203-1

**Published:** 2020-08-19

**Authors:** Yarui Ma, Qing Zhu, Junbo Liang, Yifei Li, Mo Li, Ying Zhang, Xiaobing Wang, Yixin Zeng, Yuchen Jiao

**Affiliations:** 1 State Key Laboratory of Molecular Oncology, National Cancer Center/National Clinical Research Center for Cancer/Cancer Hospital, Chinese Academy of Medical Sciences and Peking Union Medical College, 100021 Beijing, China; 2 State Key Laboratory of Medical Molecular Biology, Institute of Basic Medical Sciences, Chinese Academy of Medical Sciences, Peking Union Medical College, 10005 Beijing, China; 3 Key Laboratory of Gene Editing Screening and R&D of Digestive System Tumor Drugs, Chinese Academy of Medical Sciences and Peking Union Medical College, 100021 Beijing, China; 4 State Key Laboratory of Oncology in South China, Collaborative Innovation Center for Cancer Medicine, Sun Yat-sen University Cancer Center, Guangzhou, China; 5 Department of Clinical Laboratory, National Cancer Center/National Clinical Research Center for Cancer/Cancer Hospital, Chinese Academy of Medical Sciences and Peking Union Medical College, 100021 Beijing, China

**Keywords:** Cancer therapy, Cancer metabolism

## Abstract

Laboratory research and pharmacoepidemiology provide support for metformin as a potential antitumor agent. However, the lack of a clear understanding of the indications of metformin limits its efficacy. Here, we performed a genome-wide CRISPR knockout negative screen to identify potential targets that might synergize with metformin. Next-generation sequencing of pooled genomic DNAs isolated from surviving cells after 18 days of metformin treatment (T18) compared to those of the untreated cells at day 0 (T0) yielded candidate genes. Knockdown of a group of cyclin-dependent kinases (CDKs), including CDK1, CDK4, and CDK6, confirmed the results of the screen. Combination treatment of the CDKs inhibitor abemaciclib with metformin profoundly inhibited tumor viability in vitro and in vivo. Although cell cycle parameters were not further altered under the combination treatment, investigation of the metabolome revealed significant changes in cell metabolism, especially with regard to fatty acid oxidation, the tricarboxylic acid cycle and aspartate metabolism. Such changes appeared to be mediated through inhibition of the mTOR pathway. Collectively, our study suggests that the combination of CDKs inhibitor with metformin could be recognized as a potential therapy in future clinical applications.

## Introduction

Metformin is an oral biguanide drug that has been widely used to treat type II diabetes.^1^ Recently, laboratory research and epidemiological studies have indicated that metformin has therapeutic potential in the prevention and/or treatment of cancer. Several cohort studies have revealed an association of metformin with a significant reduction in cancer mortality.^2–4^ Moreover, emerging laboratory findings support the effectiveness of metformin in the treatment of cancer. Metformin inhibits the proliferation of hepatocellular carcinoma (HCC) cells through alteration of microRNAs, leading to G1 cell cycle arrest.^5^ In other studies, metformin suppressed human head and neck squamous cell carcinoma through global inhibition of protein translation.^6^

There is increasing interest in therapeutically exploiting the potential indications of metformin in oncology due to its safety and accessibility, as well as its significant sensitization to clinical chemotherapeutic drugs. The combination of metformin with low-dose doxorubicin has comparable effects on tumor regression and preventing relapse in vitro and in vivo, although neither is effective as a single therapy.^7^ In addition, metformin enhanced the sensitivity of endometrial cells to cisplatin and paclitaxel through repression of GLO1 expression.^8^ Finally, metformin may protect against cisplatin-induced toxicity.^9^

Based on these observations, metformin has been gradually introduced into the clinic as an adjuvant treatment for cancer. However, the indications and the mechanisms underlying its antitumor activities remain unspecified, inhibiting its use. To comprehensively elucidate the indications for metformin in the treatment of human cancer, we performed a genome-wide CRISPR knockout negative screen to identify potential gene targets that might synergize with metformin. The strategy yielded gene targets for which small molecule inhibitors have already been developed and are in clinical trials. Using these tools, we elucidated a mechanism of how combination treatment might work. Thus, these data provide a rationale for the use of combination treatment in cancer.

## Materials and methods

### Ethics statement

All animal experiments were approved by and performed according to the animal ethical committee at the Cancer Hospital, Chinese Academy of Medical Sciences (Beijing, China).

### Cell lines and compounds

Cell lines were obtained from the National Infrastructure of Cell Line Resource (Shanghai, China). U251 and HEK293T cell lines were cultured in DMEM. The MCF7 cell line was cultured in DMEM with 10 mM HEPES, 1 mM sodium pyruvate (Sigma; St. Louis, MO, USA), and 200 mM l-glutamine (Gibco/Thermo Fisher Scientific, Waltham, MA, USA). The A549 cell line was cultured in RPMI 1640. All media contained 10% fetal bovine serum and penicillin (100 U/mL)/streptomycin (0.1 mg/mL). All cells were cultured at 37 °C in a humidified atmosphere containing 5% CO_2_.

Metformin and abemaciclib were purchased from MedChemExpress (Beijing, China). The CCK8 reagent was purchased from Dojindo (Beijing, China).

### Pooled library amplification and viral production

The pooled plasmid Brunello library (RRID: Addgene_73179) was amplified according to the instructions on the Addgene website. HEK293T cells were plated and transfected 24 h later with the Brunello library (21 μg), psPAX2 (15 μg; RRID: Addgene_12260) and pMD2.G (6 μg; RRID: Addgene_12259) using the transfection reagent Neofect^TM^ (Neo Biotech; Beijing, China). The medium was aspirated after 12 h and replaced with fresh DMEM containing 10% FBS. The supernatant containing virus was harvested after 60 h, centrifuged at 104 rcf for 10 min, filtered through a 0.45 μm PVDF membrane (Millipore; Burlington, MA, USA), aliquoted and frozen at −80 °C for storage.

### CRISPR screening study

For the negative screen, the concentration (IC10 = 10 mM) of metformin required to inhibit the proliferation of U251 cells in a 15-cm plate was determined through cell counting after different dosages of metformin treatment for 48 h (Supplementary Fig. S1), and a total of 130 × 10^6^ U251 cells (500-fold coverage of the Brunello library) was transduced with the lentivirus library (multiplicity of infection = 0.3) and selected in puromycin (2 μg/mL) for 2 days. Surviving cells (3.822 × 10^7^ cells) were treated with 10 mM metformin. Cells were passaged every 3 days and treated with metformin for 18 days. DNA was extracted from T0 and T18 cells with metformin treatment using the Blood & Cell Culture DNA Maxi Kit (Qiagen, #13362; Germantown, MD, USA). The sgRNA sequences were amplified with flanking primers using Q5 Hot Start HiFi PCR Master Mix (NEB, #M0543L; Ipswich, MA, USA) and prepared for next-generation sequencing through two rounds of PCR. In the first round, 3 μg of DNA template was used (250-fold coverage of sgRNA counts) to amplify the 20 bp sgRNA cassette in 20 reactions. In the second round, Illumina-sequencing adapters and barcodes were attached through 10 cycles of PCR. The PCR products were purified with AMPure XP beads (Beckman Coulter; Brea, CA, USA) and quantified on a Qubit/Bioanalyzer 2100 (Agilent; Santa Clara, CA, USA). The diluted PCR libraries were sequenced on a HiSeq X10 platform (Illumina; San Diego, CA, USA). The primers used in the two rounds of PCR are listed in Supplementary Table S1.

### CRISPR screening data analysis

The sgRNA sequence was selected from the paired end sequence files using the FASTX-Toolkit (http://hannonlab.cshl.edu/fastx_toolkit/index.html). The sgRNA reads were mapped to the GeCKOv2 sgRNA library with Bowtie (version 1.1.2).^10^ For each sgRNA in the library, the count of mapped reads was calculated for each time point, T0 or T18. A maximum-likelihood estimation (MLE) of the gene essentiality score for each gene was generated using the Model-based Analysis of Genome-wide CRISPR/Cas9 Knockout (MAGeCK) algorithm.^11^ Candidate genes synergistic with metformin were selected for further analysis based on differences between T0 and T18 in the metformin treatment groups with *P*-values of <0.05 and filtration of essential genes of the U251 cell line (Supplementary Table S2).

### RNA interference

U251, A549, and MCF-7 cells were transfected with *CDK1, CDK4, CDK6*, or *MTOR* siRNAs (50 nM final concentration; GenePharma; Suzhou, China) using Lipofectamine RNAiMAX (Invitrogen/Thermo Fisher Scientific) according to the manufacturer’s protocols. The siRNA sequences used are listed in Supplementary Table S3.

### Cell viability assay

Cells were seeded in triplicate in 96-well plates at a density of 4000 cells/well. After 24 h, the cells were exposed to various concentrations of drug for 48 h. CCK8 (10 μL) was added to 100 μL of medium per well and incubated with the cells for 1 h at 37 °C and 5% CO_2_. The absorbance at 450 nm was detected using a microplate reader (Bio-Rad Laboratories; Hercules, CA, USA). The data were analyzed with GraphPad Prism software version 6.00 for Windows (La Jolla, CA). The combination index was calculated with CompuSyn software (ComboSyn, Inc., Paramus, NJ 07652, USA) according to the manufacturer’s instructions. Each experiment was repeated three times.

### Flow cytometric analysis

Cells were treated with drugs at the indicated concentrations for 48 h. The Annexin V Apoptosis Detection Kit (Dojindo; Kumamoto, Japan) was used to detect apoptotic cells according to the manufacturer’s instructions. Cell cycle analysis was performed after fixation in 70% ethanol overnight and incubation with propidium iodide (Beyotime; Haimen, China). All samples were analyzed on a flow cytometer (Beckman Coulter). FlowJo and Modfit LT 3.2 (Verity Software House; www.vsh.com; Topsham, ME) were used for data and cell cycle analysis.

### Metabolomics profiling

U251 cells were cultured in 10-cm dishes in DMEM overnight and then treated with DMSO, metformin (5 mM), abemaciclib (1.25 μM), or the combination of metformin/abemaciclib for 48 h. Cells were rinsed with PBS, and metabolites were extracted with prechilled 80% (v/v) HPLC-grade methanol. After centrifugation, the supernatant containing the metabolites was added to a new tube for concentration in a vacuum centrifugal concentrator for 1 h. The metabolites were redissolved proportionally according to the protein concentration measured using precipitation before liquid chromatography–tandem mass spectrometry/mass spectrometry (LC–MS/MS) analysis (Supplementary Table S4). LC separation was performed on an Ultimate 3000 UHPLC (Dionex) coupled with a Q Exactive^TM^ (Thermo Fisher Scientific). Data for metabolomics were acquired in both positive (ESI+) and negative (ESI−) modes. The mobile phases in different ionization modes were previously described.^12^

All identified polar metabolites were used in statistical analysis. The SIMCA15.0.2 software package (Sartorius Stedim Data Analytics AB; Imea, Sweden) was used for multivariate analysis and principal component analysis (PCA). Supervised orthogonal projections to latent structures discriminant analysis (OPLS-DA) was performed to visualize group separation and find significantly changed metabolites. The variable importance in the projection (VIP) score of the first principal component in OPLS-DA analysis was obtained. The metabolites with a VIP > 1 and *P* < 0.05 (Student’s *t* test) were considered to be significantly changed metabolites. A chord plot^13^ was constructed by calculating the Spearman rank correlation coefficient (*r*) for the variables with the values above a threshold. Pathway analysis was performed using MetaboAnalyst (www.metaboanalyst.ca).

### Western blot analysis

Cell lysates were extracted from cells using RIPA lysis buffer. Proteins were separated by SDS–PAGE (10% polyacrylamide), transferred to a PVDF membrane (Millipore), and blocked in 5% dry milk in Tris-buffered saline with Tween 20 (TBST; Applygen Technologies, Beijing, China). Membranes were incubated with primary antibody, and target proteins were detected with enhanced chemiluminescence (ECL) using ECL detection reagent (Applygen Technologies, Beijing, China). Primary antibodies included the following: AKT (Cell Signaling Technology Cat# 9272), phospho-AKT (Cell Signaling Technology Cat# 4060), AMPKα (D5A2) (Cell Signaling Technology Cat# 5831), phospho-AMPKα (Thr172) (40H9) (Cell Signaling Technology Cat# 2535), mTOR (Cell Signaling Technology Cat# 2983), phospho-mTOR (Abcam Cat# ab109268), p70S6K (Proteintech Cat# 14485-1-AP), phospho-p70S6K (Cell Signaling Technology Cat# 9234), 4eBP1 (Proteintech Cat# 60246-1-Ig), phospho-4eBP1 (Cell Signaling Technology Cat# 2855), GAPDH (Proteintech Cat# 60004-1-Ig), CDK1 (Santa Cruz Biotechnology Cat# sc53219), CDK4 (Cell Signaling Technology Cat# 12790), CDK6 (Abcam Cat# ab124821), Rb (Cell Signaling Technology Cat# 9309), and phospho-Rb (Ser795) (Cell Signaling Technology Cat# 9301).

### Animal studies

Five-week-old male Balb/c nude mice were subcutaneously inoculated with 5 × 10^6^ U251 cells suspended in 100 μL of PBS. Caliper measurements were made every 2 days to determine the tumor size, which was calculated using the following ellipsoid formula: (*a* × *b*^2^)/2, where *a* is the large diameter of the tumor and *b* is the small diameter. The investigator was blinded to group allocation or when experimental outcome was assessed.

### Immunohistochemistry (IHC)

An indirect peroxidase method was used for immunohistochemistry. Paraffin-embedded tumor tissues on slides were dewaxed and hydrated. All tissues were incubated overnight with primary antibody at 4 °C. Then, the tissues were incubated with goat anti-rabbit IgG (ZSGB-BIO CAS#PV-9001, Beijing, China) at room temperature. Diaminobenzidine (DAB, ZSGB-BIO, Beijing, China) was used as the chromogenic substrate.

### Statistical analysis

GraphPad Prism software version 6.00 for Windows was used for data analysis. Data are shown as the mean ± standard deviation (SD). Two-way ANOVA was used to compare the cell viability and the metabolites from different groups. *P < 0.01, **P < 0.001, ***P < 0.0001, and ****P < 0.00001.

## Results

### Genome-scale CRISPR screen identifies synergistic partners of metformin

**Fig. 1:**
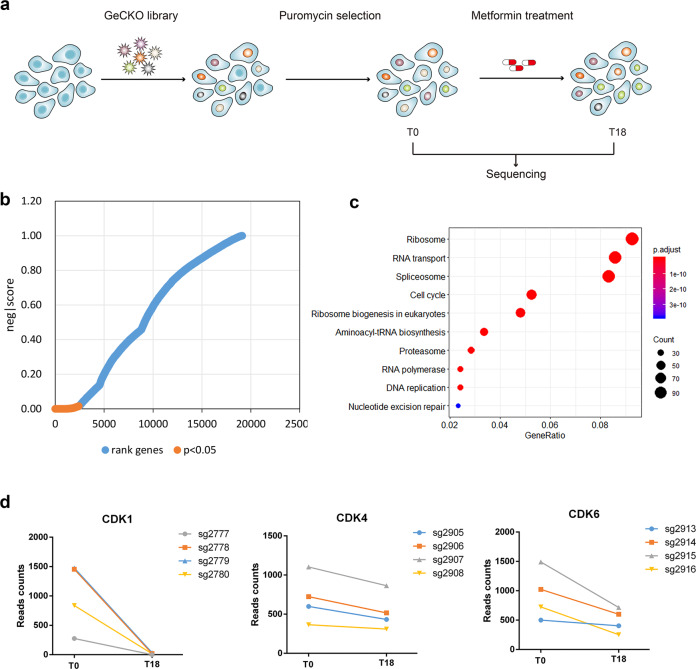
A genome-wide CRISPR/Cas9 screen identifies vulnerabilities to metformin in U251 cells. **a** Schematic of genome-wide CRISPR/Cas9 screening for candidate genes that are synergistic with metformin. **b** The scores for each gene in the control (*T*0) and metformin (*T*18) treatment groups. Orange dots represent the genes synergistic with metformin. **c** Pathway enrichment analysis of the genes identified in the screen using the KEGG database. **d** Differences in sgRNA counts for *CDK1*, *CDK4*, and *CDK6* between *T*0 and *T*18

To identify genes whose loss might sensitize cells to metformin treatment, we conducted a genome-wide CRISPR-Cas9 loss-of-function screen in the U251 cell line. Our overall strategy was to sequence sgRNAs obtained from the remaining viable cells at two time points, *T*0 and *T*18 (after 18 days under metformin treatment), to identify candidate synergistic genes. We expected that sgRNAs targeting genes synergistic with metformin would be depleted at *T*18 compared with *T*0. We transfected cells with the Brunello library containing 76,441 guides with ~4 guides per gene. Sufficient cell numbers (3.822 × 10^7^ cells) were transduced to allow 500× coverage of each sgRNA within the library. This level of coverage was maintained at each cell passage. U251 cells were selected for stable viral integration in puromycin for 2 days and then treated with low-level (IC10 = 10 mM determined by cell counting in 15-cm plates) metformin for 18 days. Deep sequencing was performed on the sgRNAs in DNA isolated from viable cells collected at *T*0 and *T*18, and comparisons were performed to determine which sgRNAs had been lost at *T*18 (Fig. 1a). Using the MAGeCK algorithm, we identified a set of 1793 fitness genes (*P* < 0.05) required for the viability of U251 cells under treatment with metformin (Fig. 1b and Supplementary Table S5).

We also performed KEGG analysis to associate genes with a putative function and found the depleted genes to be involved in the ribosome, the spliceosome, the cell cycle, RNA transport, and DNA replication (Fig. 1c). We focused on a set of cyclin-dependent kinases (CDKs), including CDK6. Given that CDK4 always functions with CDK6 and CDK1 is one of the most important genes in the cell cycle, we added CDK1 and CDK4 in the following functional assays, as they are druggable targets with several inhibitors already under clinical investigation (Fig. 1d).

### A combination of CDK knockdown with metformin significantly promotes tumor inhibition in vitro

**Fig. 2:**
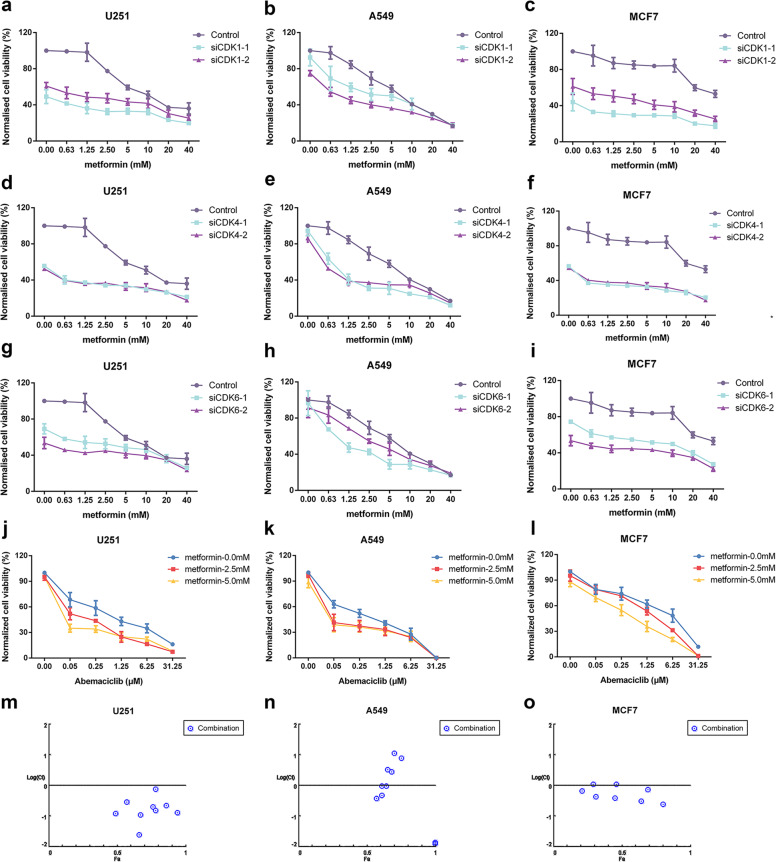
Inhibition of CDK4/6 potentiates the antiproliferative effects of metformin in vitro. **a**–**c** Cell viability measured using the CCK8 assay after siRNA knockdown of CDK1 in U251, A549, and MCF7 cells in the absence or presence of metformin at multiple concentrations for 48 h. **d**–**f** Cell viability measured using the CCK8 assay in U251, A549, and MCF7 cells with siRNA knockdown of CDK4 in the absence or presence of metformin at multiple concentrations for 48 h. **g**–**i** Cell viability measured using the CCK8 assay after siRNA knockdown of CDK6 in U251, A549, and MCF7 cells in the absence or presence of metformin at multiple concentrations for 48 h. **j**–**l** U251, A549, and MCF7 cells treated with different doses of metformin and abemaciclib for 48 h. **m**–**o** The CI calculated using CompuSyn software, where CI <1, =1, and >1 indicate synergism, additive effect and antagonism, respectively. Cell viability was measured using the CCK8 assay. One representative experiment of three is shown with *n* = 3 ± SD for each data point. *P < 0.01, **P < 0.001 (two-way ANOVA, three technical replicates averaged in each)

To evaluate whether CDK1/CDK4/CDK6 expression was associated with metformin sensitivity, we knocked down each of these CDKs independently in U251 cells using RNA interference (Supplementary Fig. S2). Knockdown of CDK1/CDK4/CDK6 with either of two siRNAs enhanced the antiproliferative activity of metformin in U251 cells by >20% compared with that of metformin treatment alone (Fig. 2a, d, g). To test the generality of the synergy, we knocked down CDK1/CDK4/CDK6 in cell lines derived from different cancer types, including A549 (non-small cell lung cancer) and MCF7 (ER^+^ breast cancer), and treated them with metformin. Cell viability was also reduced in these cell lines by ≥~20%. Compared to the metformin group or CDK knockdown group, the CDK knockdown/metformin group had a significant difference at most concentrations of metformin (Supplementary Fig. S2; Fig. 2b, c, e, f, h, i; Supplementary Tables S6–S14). Therefore, a combination of CDK knockdown with metformin significantly promoted tumor inhibition in vitro.

### Metformin shows synergistic antiproliferative effects with the CDKs inhibitor abemaciclib in different cancer cell lines

Some small molecule inhibitors of CDKs, such as abemaciclib, have been developed and are already under clinical investigation. Abemaciclib is a novel inhibitor that is selective for CDK4/6. Therefore, we examined whether abemaciclib combined with metformin is also synergistic in suppressing the growth of cancer cell lines. The IC50 of abemaciclib was in the range of 0.29–2.40 μM in different cell lines, demonstrating a greater potency in inhibiting cell growth than metformin. Cotreatment of metformin with abemaciclib in U251, A549, and MCF7 cell lines further reduced cell viability compared with either treatment alone (Fig. 2j–l). There was a significant difference between the metformin/abemaciclib group and the abemaciclib group or metformin group at most of the metformin concentrations (Supplementary Tables S15–S17). Next, we calculated the combination index (CI), which is defined as synergistic for a value of <1 and as antagonistic if >1, of the abemaciclib and metformin interaction in CompuSyn software using the Chou–Talalay method.^14^ The CI values for all three cell lines tended to be <1 and thus revealed synergistic antiproliferative effects (Fig. 2m–o). These results indicated that cotreatment of cells with metformin and abemaciclib reduced cell viability.

### Apoptosis or cell cycle arrest does not mediate the synergy induced by combination treatment

**Fig. 3:**
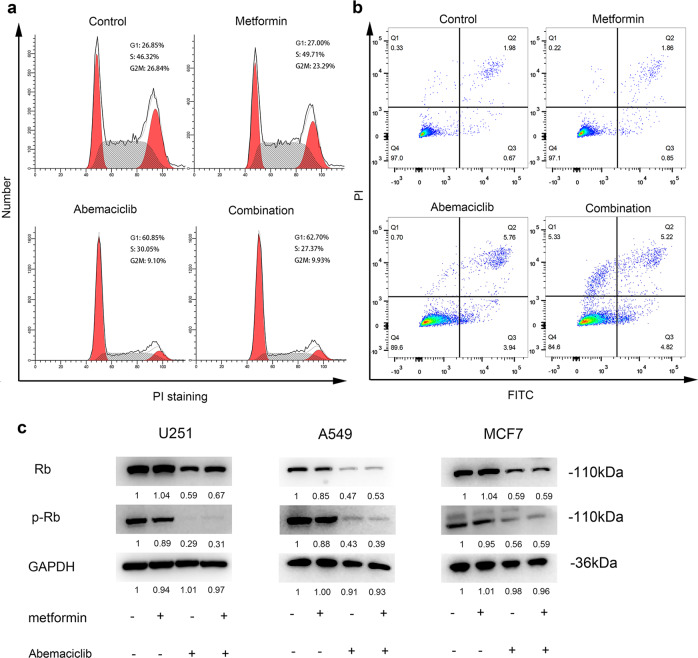
The synergistic effect caused by metformin/abemaciclib is independent of apoptosis and cell cycle arrest. **a** Cell cycle distribution in the control, metformin-treated (5 mM), abemaciclib-treated (1.25 µM), and combination-treated U251 cells for 48 h. **b** Flow cytometry showing Annexin V staining of U251 cells exposed to metformin (5 mM), abemaciclib (1.25 μM) and metformin/abemaciclib for 48 h. **c** Western blots of protein lysates prepared from U251, A549, and MCF7 cells treated with metformin (5 mM), abemaciclib (1.25 µM), and metformin/abemaciclib for 48 h

Both metformin and CDK4/6 inhibitors have been shown to induce cell cycle arrest. Metformin causes G1 cell cycle arrest in human hepatocellular carcinoma (HCC) cells,^5^ while CDK4/6 inhibitors tend to inhibit the transition from G1 to S.^15^ We therefore evaluated cell cycle progression in treated U251 cells with flow cytometry. The cell cycle parameters did not change significantly under metformin treatment. The discrepancy with previous reports could derive from cell type-specific effects.^16^ Moreover, we found that abemaciclib induced G1 arrest in the U251 cell line (abemaciclib vs. controls: G1: 60.85% vs. 26.85%; S: 30.05% vs. 46.32%; G2M: 9.10% vs. 26.84%; Fig. 3a). The combination treatment did not significantly alter the cell cycle parameters compared to abemaciclib treatment alone (G1: 60.85% vs. 62.70%; S: 30.05% vs. 27.37%; G2M: 9.10% vs. 9.93%). Abemaciclib either alone or in combination with metformin led to a much higher proportion of apoptotic cells than those in the metformin and control groups. However, we observed no significant differences between the abemaciclib and combination treatment groups (Fig. 3b).

The results from Western blot analysis were consistent with the changes observed in the cell cycle parameters under the various treatments. Phospho-Rb remained unchanged in response to metformin but was reduced significantly by abemaciclib (~2–3×). However, the protein was not further reduced under the combination treatment (Fig. 3c). These data suggested that alterations in apoptosis and cell cycle parameters do not fully explain the synergistic interaction of metformin and abemaciclib.

### Metformin and CDKs inhibitor alter the metabolome of cell lines

**Fig. 4:**
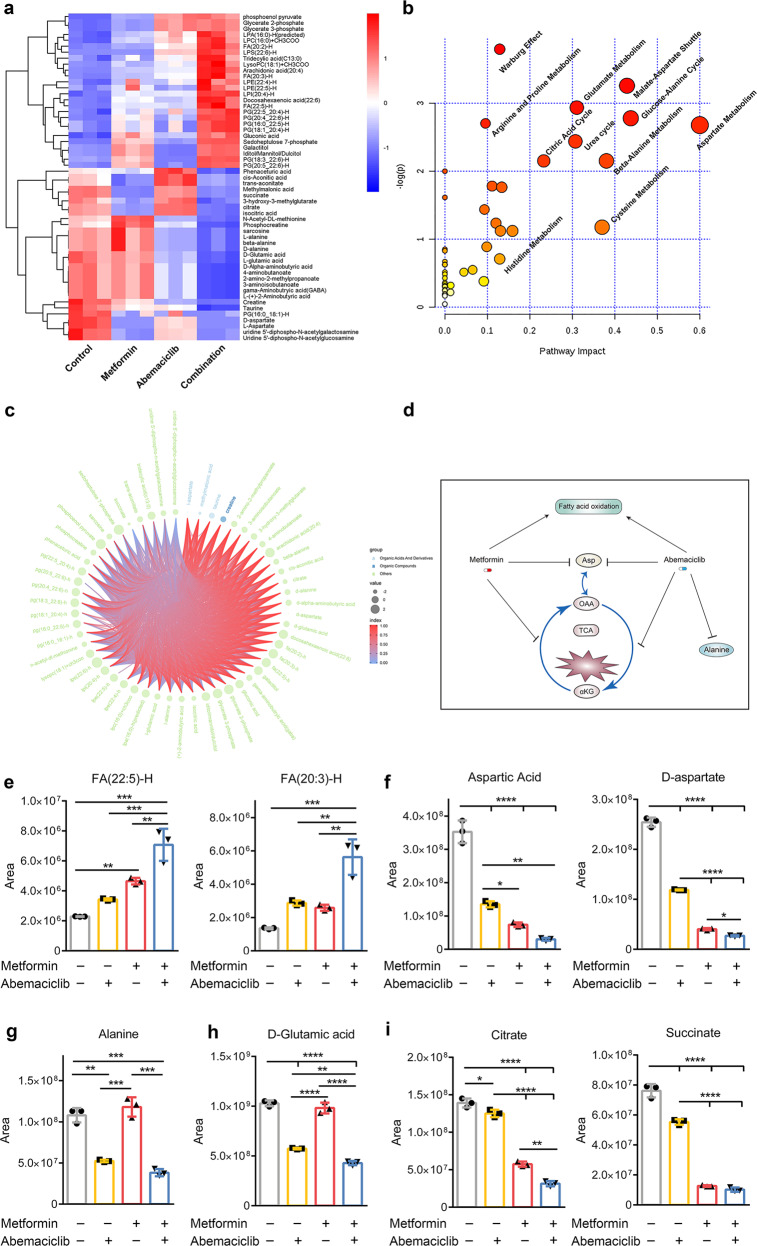
The combination treatment with metformin and abemaciclib changed the metabolome of cancer cells. **a** A heat map of differentially abundant metabolites obtained from the control, metformin (5 mM), abemaciclib (1.25 µM), and combination-treated groups at 48 h in the negative (ESI−) ion mode. **b** Pathway enrichment analysis of differentially abundant metabolites of all groups using MetaboAnalyst 4.0 website in the negative (ESI−) ion mode. **c** Chord plots generated using the Spearman rank correlations for the differentially abundant metabolites of the four groups in the negative (ESI−) ion mode. **d** Schematic of the altered metabolic pathways, including the TCA cycle, aspartate metabolism, and fatty acid oxidation, underlying the synergistic efficacy of metformin and abemaciclib. **e**–**i** Levels of the indicated metabolites in U251 cells treated with metformin (5 mM), abemaciclib (1.25 µM), or the combination for 48 h as determined by LC–MS/MS. Data are shown as the mean ± SD. *P < 0.01, **P < 0.001, ***P < 0.0001, and ****P < 0.00001 (two-way ANOVA; three technical replicates averaged in each)

Growing evidence demonstrates that CDK4 is a “metabolic” cell-cycle regulator^17,18^ and is involved in fatty acid metabolism,^19^ while metformin changes the metabolism of mitochondria through inhibition of respiratory-chain complex 1.^1^ Therefore, we explored whether the synergy of the combination treatment was due to changes in the metabolome of cancer cells. We performed metabolic profiling on U251 cells treated for 48 h as indicated: control; metformin, 5 mM; abemaciclib, 1.25 μM; combination, metformin, 5 mM, and abemaciclib, 1.25 μM. A total of 26 and 30 metabolites were significantly upregulated in the negative and positive ion modes, respectively (VIP > 1, *P* < 0.05, ratio of the combination and control groups >1.5), while 20 and 5 metabolites were downregulated in the negative and positive ion modes, respectively (VIP > 1, *P* < 0.05, ratio of the combination and control groups <0.75; Fig. 4a; Supplementary Fig. S3). These metabolites were uploaded for analysis using the online algorithm MetaboAnalyst 4.0 to identify the key metabolic pathways involved (Fig. 4b and Supplementary Fig. S3). Spearman rank correlations were also generated for these metabolites and visualized in a chord plot (Fig. 4c). This approach further revealed the interactions of these metabolites. We found that aspartate, citric acid, and glutamate metabolism were decreased, while beta oxidation of very long chain fatty acids was increased.

Long-chain fatty acids, such as FA(20:3)-H and FA(22:5)-H, were dramatically upregulated after combination treatment relative to either drug treatment alone (Fig. 4e). These results suggested that long-chain fatty acid oxidation was enhanced under the combination treatment. The combination treatment also significantly decreased l-aspartic acid, d-aspartic acid, succinic acid, l-alanine, and l-glutamic acid, which are associated with the aspartate and glutamate metabolic pathways (Fig. 4f–h). Moreover, the levels of succinic acid, isocitric acid, *cis*-aconitic acid, and citric acid, which all play a key role in the citric acid cycle (the TCA cycle), were decreased (Fig. 4i). Furthermore, several other metabolic pathways involving glycine, arginine, and proline were altered. These data indicated that metformin and abemaciclib affect the cancer metabolome by promoting fatty acid oxidation, inhibiting aspartate and suppressing the TCA cycle (Fig. 4d). Thus, such changes might be sufficient to inhibit tumor cell proliferation under the combination treatment.

### Metformin and the CDKs inhibitor cooperatively inhibit mTOR

**Fig. 5:**
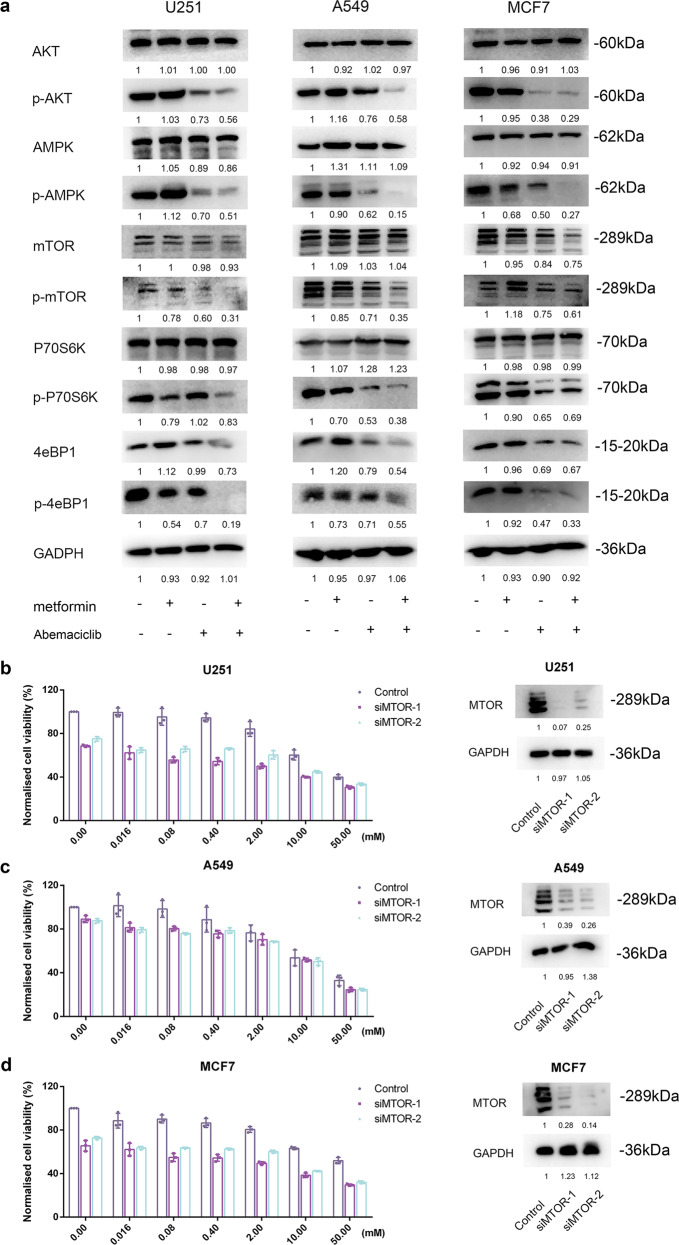
The combination treatment is mediated by changes in mTOR. **a** Western blots to detect expression levels and the phosphorylation status of AMPK-associated and mTOR-associated proteins using protein lysates prepared from U251, A549, and MCF7 cells treated with metformin (5 mM), abemaciclib (1.25 µM), or the combination for 48 h. **b** U251, **c** A549, and **d** MCF7 cells were treated with or without different concentrations of metformin for 48 h after knockdown of mTOR. Data are shown as the mean ± SD. *P < 0.01, **P < 0.001 (two-way ANOVA, three technical replicates averaged in each). mTOR expression after knockdown of mTOR through RNA interference in U251, A549, and MCF7 cells

To identify molecular mechanisms that might underlie the changes in the metabolome under combination treatment, we performed Western blot analyses on protein lysates prepared from treated U251, A549, and MCF7 cells. Treatment with abemaciclib alone led to reduced levels of p-AKT, p-AMPK, p-mTOR, p-P70S6K, 4eBP1, and p-4eBP1 in all three cell lines, and cotreatment led to a further reduction in the phosphorylation levels (Fig. 5a). However, no treatment regimen affected the protein levels of AKT, AMPK, and P70S6K. Among these proteins, mTOR downregulation may be associated with alterations in the metabolome according to our Western blot results and previous reports.^20^ Next, we assessed whether mTOR inhibition was involved in the synergetic effect of metformin and CDKs inhibitor. Several concentrations of metformin/mTOR knockdown could kill more cells than those in the mTOR knockdown group or metformin group, indicating that mTOR inhibition could be involved in the synergistic mechanism for metformin (Fig. 5b–d; Supplementary Tables S18–S20). Nevertheless, the trend decreased with high concentrations of metformin as toxic and side effects increased correspondingly. Taken together, these results indicated that the synergistic effect of the combination treatment was due to alterations in the metabolome caused by inhibition of the mTOR pathway rather than cell cycle arrest.

### The combination treatment suppresses tumor growth in vivo

**Fig. 6:**
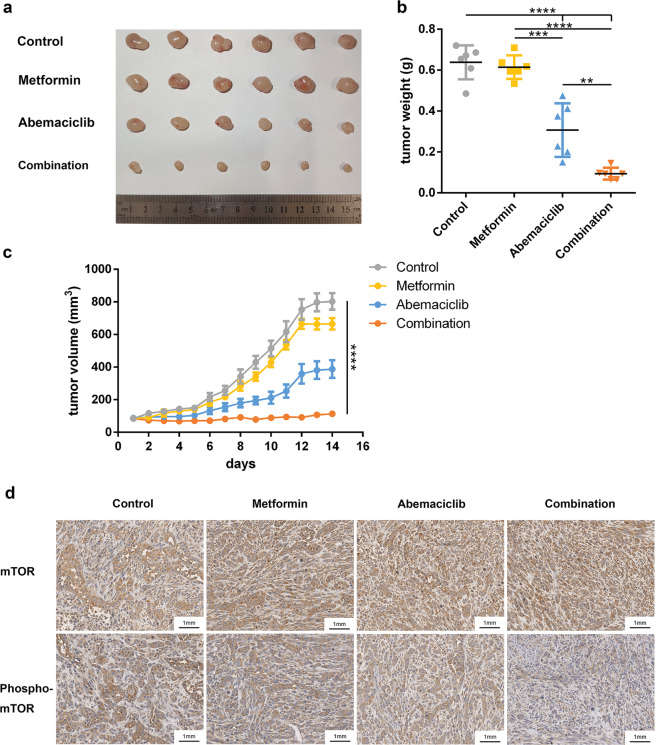
The combination treatment suppresses tumor growth in an in vivo xenograft model. U251 cells were injected subcutaneously, and after the tumor volumes reached 100 mm^3^, mice were divided into the following treatment groups (*n* = 6 animals/group): vehicle, metformin (100 mg/kg), abemaciclib (45 mg/kg), or the combination of metformin and abemaciclib. Animals were treated every day for 14 days by oral gavage. The data are presented as the mean ± SEM, **P < 0.001, ***P < 0.0001, and ****P < 0.00001 (two-way ANOVA). **a** Representative images of xenograft tumors. **b** Weights of xenograft tumors after dissection. **c** Plots of tumor volumes over 14 days of treatment. **d** Immunohistochemical staining with mTOR and phospho-mTOR antibodies was performed on the xenografts

To assess the efficacy of the combination treatment in vivo, we evaluated tumor growth in a subcutaneous xenograft model. Xenograft tumors were generated through subcutaneous injection of U251 cells into nude mice. Once the tumor volumes reached between 100 and 120 mm^3^, the mice (*n* = 6) were randomized into four groups and administered vehicle, metformin, abemaciclib, or the combination of metformin and abemaciclib by oral gavage every day. After 14 days, the tumor volumes increased in the animals treated with vehicle, metformin, or abemaciclib, but remained largely unchanged under the combination treatment (Fig. 6a–c). Abemaciclib and the combination treatment suppressed tumor growth by >~2× relative to the vehicle control. Moreover, phospho-mTOR expression was dramatically decreased compared to that of the other groups, while mTOR expression was not obviously changed (Fig. 6d and Supplementary Fig. S4). Taken together, these results demonstrated that the combination treatment of metformin with abemaciclib profoundly inhibited tumor growth in vivo.

## Discussion

Metformin has emerged as a promising agent for cancer treatment and prevention in both epidemiological and molecular studies.^21^ In many retrospective population studies, for example, metformin has been associated with a reduced cancer risk and improved prognosis.^22^ These ideas have been transferred to the lab bench, and many studies have reported the growth inhibitory properties of metformin in multiple tumor cell lines, including breast, lung, liver, and stomach tumor cell lines.^23^ In this study, we used a genome-wide CRISPR knockout screen to identify genes whose loss might synergize with metformin in the GBM cell line U251. We identified sgRNAs targeting genes associated with the cell cycle (*CDK6*) and subsequently demonstrated that the synergy of metformin and small molecule inhibitors of CDK4/6 was associated with aberrant metabolism potentially mediated through the inhibition of mTOR.

We focused on CDK1/CDK4/CDK6 given that increased expression of CDKs has been associated with many tumor types, and oral, highly selective CDKs inhibitor have already been developed. CDK1/CDK4/CDK6 inhibitors, such as abemaciclib and palbociclib, represent an important therapeutic advancement in oncology and have shown promising anticancer activity and manageable toxicity in clinical trials.^24–26^ CDKs emerged from our CRISPR screen as candidate genes that, if knocked out, might synergize with metformin as an antitumor therapy. Using a siRNA knockdown approach in combination with metformin treatment, we found that loss of CDK1/CDK4/CDK6 in combination with metformin exhibited enhanced antitumor properties in several tumor cell lines. Moreover, different concentrations may affect the synergistic interaction, which inspired us to search for optimal synergistic dose combinations for tumor management in the future.^27^ Finally, we demonstrated that the combined treatment of abemaciclib and metformin profoundly inhibited cancer cell viability in vitro and in vivo.

Metformin has been reported to activate AMPK through modest inhibition of Complex I and inhibit mTOR, which regulates several biosynthetic pathways.^28–31^ In addition, aberrations in the cyclin D-CDK4/6 axis occur commonly in diverse cancers, including head and neck cancer,^32^ breast cancer,^33^ non-small-cell lung cancer,^34^ esophageal cancer,^35^ melanoma,^36^ and glioblastoma.^15,37^ CDK4/6 are serine/threonine kinases that are associated with D-type cyclins and promote G1 phase progression in preparation for DNA synthesis.^38^ Abemaciclib inhibits CDK4/6 and reduces the phosphorylation of RB1, thereby inducing G1 arrest and growth regression.^39^ Here, we investigated whether the synergy between metformin and abemaciclib was due to enhanced cell cycle arrest or apoptosis. Flow cytometry experiments, however, demonstrated that cell cycle parameters were not significantly altered under the combination treatment relative to abemaciclib treatment alone. These data suggested that cell cycle arrest is insufficient to explain the synergy of metformin and CDKs inhibitor even though abemaciclib can effectively interrupt the cell cycle.

Since the mechanisms for synergistic growth suppression did not involve changes in the cell cycle or apoptosis, we explored the possibility of alterations in the metabolome using LC–MS/MS. We provided evidence suggesting that the aberrant metabolome was essential for the synergistic interaction between metformin and CDKs inhibitor. The metabolomic profile of metformin/abemaciclib-treated tumors revealed a dramatic increase in several metabolites associated with fatty acid oxidation metabolism. On the one hand, it is not expected that CDK4 would phosphorylate and repress the α2 subunit of AMPK, the major regulator of oxidative metabolism.^40^ Additionally, CDK4 creates a positive feedback loop that maintains adipocyte insulin signaling through phosphorylation of insulin receptor substrate 2 at serine 388.^18^ On the other hand, CDK4 appears to promote anabolism by blocking catabolic processes (fatty acid oxidation), which are activated by AMPK.^19,41^ Here, our data showed that very long chain fatty acids were increased after treatment with abemaciclib. AMPK was, however, not activated, indicating that CDK4 might repress fatty acid oxidation through multiple pathways, including mTOR inhibition.

Metabolome profiling also showed that the aspartate and TCA metabolic pathways were decreased dramatically under the combination treatment relative to treatment with each drug alone. Aspartate is a limiting metabolite for cancer cell proliferation.^42,43^ We also showed that the tricarboxylic acid cycle was disturbed by metformin/abemaciclib, which may restrict cell proliferation together with aspartate deficiency. These results corroborate previous findings that metformin suppresses tumor growth through aspartate limitation.^44^ Abemaciclib further reduced aspartate levels in our model systems. Such changes appear to be mediated partially through suppression of mTOR by either drug alone and enhanced through the combination treatment, which suppressed mTOR and AMPK. Thus, the mTOR pathway might mediate regulation of the metabolome under the combination treatment.

The combination treatment also appears to downregulate glutamine metabolism, which is important in supporting cell proliferation.^45^ Glutamine upregulation is a common feature among tumor types and participates in energy generation, signal transmission, and biological synthesis by providing a source of carbon and nitrogen.^45,46^ Although our data demonstrated that decreased glutamate may partly contribute to the synergy of metformin and abemaciclib, we need to validate the results in further clinical trials and molecular experiments to elucidate the precise mechanism of their synergy in the suppression of tumor growth.

In summary, we first demonstrated that the combination of metformin with a CDK4/6 inhibitor can provide a remarkable therapeutic advantage against three tumor types over either agent alone. Compared to treatment with the CDK4/6 inhibitor alone, the combination treatment of metformin with abemaciclib also exerted its cytotoxic effects through inhibition of the mTOR pathway. We uniquely approached the study of metformin by combining biochemistry, molecular biology, and in vivo experiments, and demonstrated that efficacy was reflected by alterations in the metabolome, namely, in aspartate, the TCA cycle, and fatty acid oxidation. The aberrant cancer metabolism caused by the synergy between metformin and abemaciclib could provide an attractive new treatment option for cancer.

## Supplementary information

Supplemental Tables (Excel version)

Supplemental Material File #1

## Data Availability

All the datasets are available from the corresponding author upon reasonable request.
