# Supplementary material for: A CRISPR knockout negative screen reveals synergy between CDKs inhibitor and metformin in the treatment of human cancer in vitro and in vivo
Source: Signal Transduct Target Ther. 2020 Aug 19;5:152. doi: 10.1038/s41392-020-0203-1 (PMC7434905; doi:10.1038/s41392-020-0203-1)
Supplement: Supplementary file 2 — Supplemental Material File #1 [file 41392_2020_203_MOESM2_ESM.docx]

Supplementary Materials for

A CRISPR knockout negative screen reveals synergy between CDKs inhibitor and metformin in the treatment of human cancer in vitro and in vivo

Yarui Ma^1#^, Qing Zhu^1#^, Junbo Liang^2^, Yifei Li^1^, Mo Li^1^, Ying Zhang^1^, Xiaobing Wang^1, 5*^, Yixin Zeng^3*^, Yuchen Jiao^1, 4, 5*^

Correspondence to: wangxb@cicams.ac.cn

**This PDF file includes:**

Figures. S1 to S4

**Other Supplementary Materials for this manuscript include the following:**

Tables S1 to S20 [separate file]

Figure. S1.


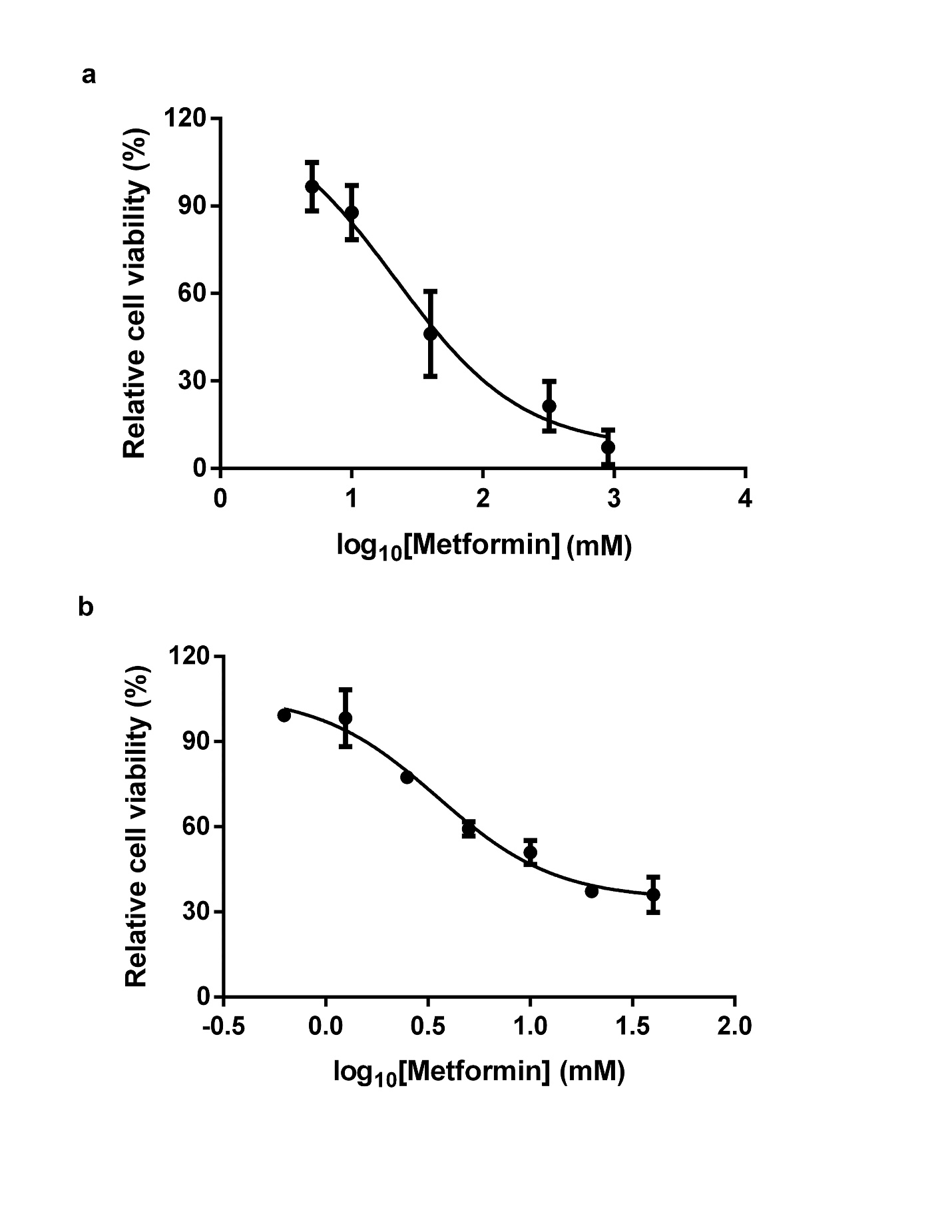
 Figure S1. Cell viability of U251 cells treated with metformin.

(a) U251 cells were treated with increasing concentrations of metformin for approximately 48 hr in 15-cm plates. Cell viability was determined by cell counting. (b) Cell viability was measured using the CCK8 assay in U251 cells treated with increasing concentrations of metformin for approximately 48 hr in 96-well plates. One representative experiment of three is shown with n = 3 ± SD for each data point.

Figure. S2.


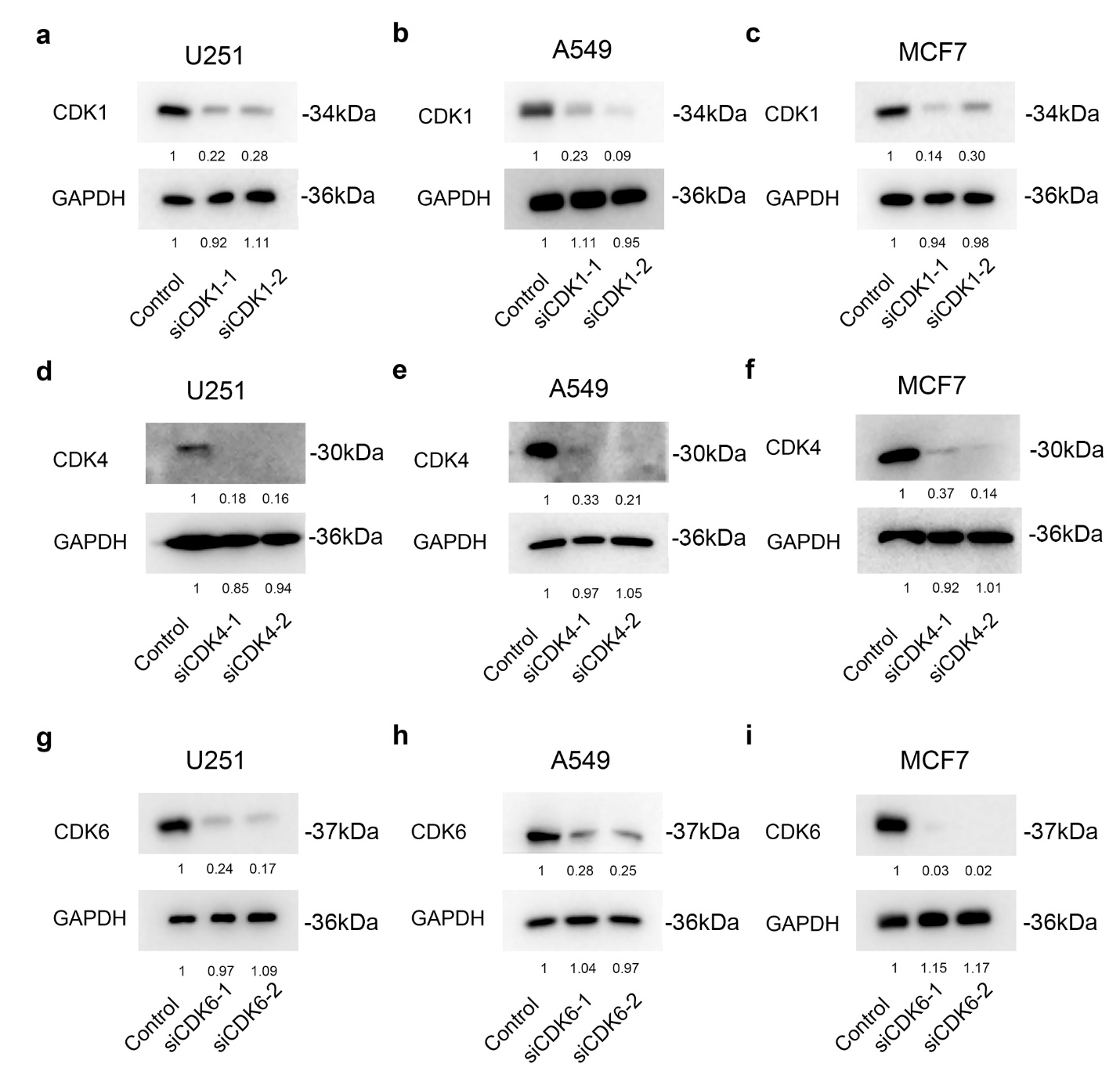
 Figure S2. siRNA confirmation assays.

(a-c) Western blots to detect the expression levels of CDK1 in U251, MCF7, and A549 cells. (d-f) Western blots to detect the expression levels of CDK4 in U251, MCF7, and A549 cells. (g-i) Western blots to detect the expression levels of CDK6 in U251, MCF7, and A549 cells.

Figure. S3.


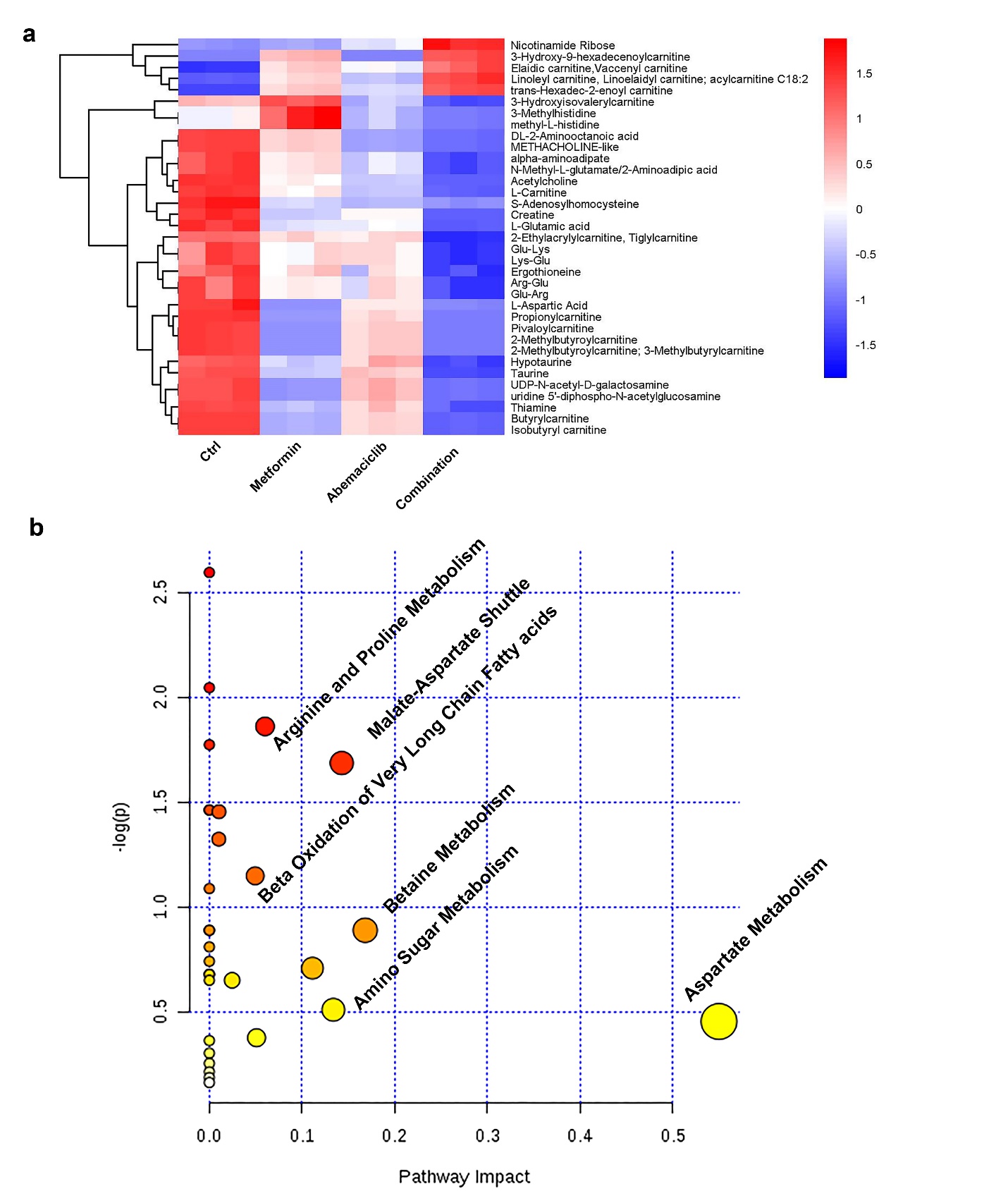
 Figure S3. Combination treatment with metformin and abemaciclib changes the metabolome of cancer cells.

(a) A heat map of differentially abundant metabolites from the control, metformin (5 mM), abemaciclib (1.25 µM) and combination groups for 48 hr obtained in the negative (ESI+) ion mode.

(b) Pathway enrichment analysis of differentially abundant metabolites from all groups obtained in the negative (ESI+) ion mode using the MetaboAnalyst 4.0 website.

Figure. S4.


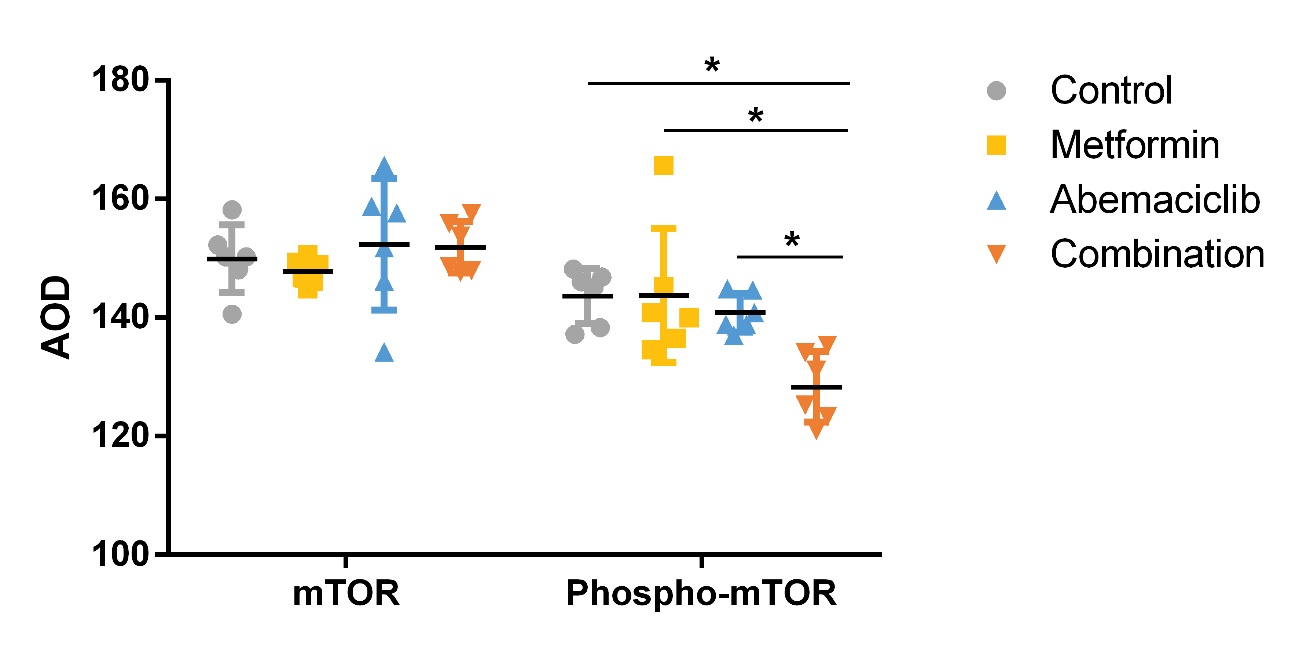


Figure S4. Quantification of mTOR and phospho-mTOR staining.

Quantification of mTOR and phospho-mTOR staining from the xenografts through ImageJ. AOD (average optical density) was used to reflect the expression of mTOR and phospho-mTOR. The data are presented as the mean ± SD, *P < 0.05 (two-way ANOVA).

Tables S1 to S20. (separate file)

| Supplementary Tables |  |
| --- | --- |
| Supplementary Table 1 | List of primers used in the two rounds of PCR |
| Supplementary Table 2 | Essential gene list for U251 cell line |
| Supplementary Table 3 | List of siRNA sequences |
| Supplementary Table 4 | The quantification of the precipitation |
| Supplementary Table 5 | Screen result |
| Supplementary Table 6 | Statistical analysis of Figure 1a |
| Supplementary Table 7 | Statistical analysis of Figure 1b |
| Supplementary Table 8 | Statistical analysis of Figure 1c |
| Supplementary Table 9 | Statistical analysis of Figure 1d |
| Supplementary Table 10 | Statistical analysis of Figure 1e |
| Supplementary Table 11 | Statistical analysis of Figure 1f |
| Supplementary Table 12 | Statistical analysis of Figure 1g |
| Supplementary Table 13 | Statistical analysis of Figure 1h |
| Supplementary Table 14 | Statistical analysis of Figure 1i |
| Supplementary Table 15 | Statistical analysis of Figure 1j |
| Supplementary Table 16 | Statistical analysis of Figure 1k |
| Supplementary Table 17 | Statistical analysis of Figure 1l |
| Supplementary Table 18 | Statistical analysis of Figure 5b |
| Supplementary Table 19 | Statistical analysis of Figure 5c |
| Supplementary Table 20 | Statistical analysis of Figure 5d |
